# Effect of Oxygen on Verbenone Conversion From *cis*-Verbenol by Gut Facultative Anaerobes of *Dendroctonus valens*

**DOI:** 10.3389/fmicb.2018.00464

**Published:** 2018-03-16

**Authors:** Qingjie Cao, Jacob D. Wickham, Li Chen, Faheem Ahmad, Min Lu, Jianghua Sun

**Affiliations:** ^1^State Key Laboratory of Integrated Management of Pest Insects and Rodents, Institute of Zoology, Chinese Academy of Sciences, Beijing, China; ^2^University of Chinese Academy of Sciences, Beijing, China; ^3^Department of Biosciences, COMSATS Institute of Information Technology, Islamabad, Pakistan

**Keywords:** oxygen environments, gut facultative anaerobes, pheromone production, *cis*-verbenol, verbenone

## Abstract

Since its introduction from North America, *Dendroctonus valens* LeConte has become a destructive forest pest in China. Although gut aerobic bacteria have been investigated and some are implicated in beetle pheromone production, little is known about the abundance and significance of facultative anaerobic bacteria in beetle gut, especially with regards to effects of oxygen on their role in pheromone production. In this study, we isolated and identified gut bacteria of *D. valens* adults in an anaerobic environment, and further compared their ability to convert *cis*-verbenol into verbenone (a multi-functional pheromone of *D. valens*) under different O_2_ concentrations. *Pantoea conspicua, Enterobacter xiangfangensis, Staphylococcus warneri* were the most frequently isolated species among the total of 10 species identified from beetle gut in anaerobic conditions. Among all isolated species, nine were capable of *cis*-verbenol to verbenone conversion, and the conversion efficiency increased with increased oxygen concentration. This O_2_-mediated conversion of *cis*-verbenol to verbenone suggests that gut facultative anaerobes of *D. valens* might play an important role in the frass, where there is higher exposure to oxygen, hence the higher verbenone production. This claim is further supported by distinctly differential oxygen concentrations between gut and frass of *D. valens* females.

## Introduction

Symbioses between insects and associated gut microbiota are the driver behind the success of the majority of the destructive pests of forests (Moran et al., 2005; Adams et al., 2011; Sun et al., 2013; Lu et al., 2016). Gut microbiota play an important role in the biology of insects including host’s nutrition, development, resistance to pathogens, and reproduction (Brand et al., 1975; Brune, 2003; Moran et al., 2005). In addition to the role in host’s nutrition (Kaufman and Klug, 1991; Breznak, 2002), microorganisms may also be involved in complex ecological functions involving pheromone production. Gut-associated microbiota are known to produce components of aggregation pheromones in locusts and sex pheromones in cockroaches (Dillon et al., 2000, 2002; Wada-Katsumata et al., 2015), influence mating preference of *Drosophila melanogaster* (Sharon et al., 2010), and mediate insect interactions with plants by degrading host defense chemicals (Mason et al., 2014) but many of these functions are performed under aerobic condition while their functions under low oxygen conditions has yet to be explored.

Bark beetles (Coleoptera: Curculionidae: Scolytinae) are among the most economically important pests of conifers causing significant losses to pine forests worldwide (Raffa and Berryman, 1987; Paine et al., 1997; Gitau et al., 2013; Wang et al., 2017; Zhou et al., 2017). Previous studies have focused on gut aerobic bacteria in major bark beetle species in order to explain the pests’ ecology (Xu et al., 2016c). For example, when exposed to α-pinene (a prominent defensive host monoterpene) in the air, the gut-associated *Bacillus cereus* isolated from *Ips paraconfusus* produced verbenol (Brand et al., 1975). Similarly, host monoterpene α-pinene has been converted to *cis*- and *trans*-verbenol, and myrtenol by bacteria isolated from the bark beetle *Ips paraconfusus* Lanier (Renwick et al., 1976). α-Pinene is a precursor in the biosynthesis of verbenone, one of the common pheromones of bark beetles (Gitau et al., 2013). These findings suggest that microorganisms of bark beetles can counteract the host plant defense molecules.

The red turpentine beetle, *Dendroctonus valens* LeConte (Scolytinae), is an aggressive, univoltine, tree-killing species and caused mortality of more than 10 million healthy pines in central areas of northern China after it first appeared in Shanxi Province in the late 1990s (Farjon and Page, 1999; Miao et al., 2001; Wang et al., 2012; Sun et al., 2013; Lou et al., 2014; Cheng et al., 2015; Zhou et al., 2016). The beetles overwinter as larvae and complete maturation the following spring. From mid-May to mid-June, adult beetles emerge and then disperse from the parent colony to new host trees through pheromone-mediated mass attack (henceforth “dispersing beetles”). Upon successful invasion of new hosts, the beetles penetrate the bark, mate and start oviposition (henceforth “colonizing beetles”). The larvae of colonizing beetles then feed on the phloem, and the cycle continues (Miao et al., 2001). Bioactive chemicals *cis*-verbenol, *trans*-verbenol, myrtenol, myrtenal, and verbenone are gut volatiles of *D. valens* identified in China (Zhang et al., 2006; Sun et al., 2013; Xu et al., 2016a). Verbenone, in general, is an aggregation pheromones of *D. valens* but its function is concentration dependent where higher concentrations may also act as an anti-aggregation pheromone (Zhang et al., 2006). The concentration of verbenone production in *D. valens* mainly depends upon the rate of conversion of *cis*-verbenol and *trans*-verbenol to verbenone. This conversion is an oxidation process, which was proven to be accelerated by gut-associated bacteria (Xu et al., 2015). However, previous studies on pheromone conversion had not taken in account the *in situ* oxygen concentration, or anything approximating that. In addition, those studies have been conducted under atmospheric O_2_ concentrations. Hence, the question stands if these findings are even valid under anaerobic or at least under very low oxygen concentrations.

Therefore, in this study we have investigated the role of gut bacteria involved in verbenone production in *D. valens* under anaerobic and low oxygen conditions. The anaerobic bacteria from the guts of *D. valens* adults in the two life history stages (dispersing and colonizing) were isolated and comparisons of their conversion efficiency of *cis*-verbenol to verbenone were made under different O_2_ concentrations. Our findings could further shed light on the role of oxygen on facultative anaerobes in verbenone production and increase understanding of the chemical ecology of red turpentine beetle and bark beetles in general.

## Experimental Procedures

### Insects and Media

*Dendroctonus valens* adults were captured from newly attacked pine trees in Beishe Mountain and Laoyagou Mountain of Shanxi province during June 2016 (N 37° 48′, E 111° 44′, average elevation 1400 m). Each sampling site was about 13 km apart and infested pine trees were randomly selected at least 200 m apart to obtain samples of dispersing adult males and females, and colonizing adult males and females.

For the preparation of anaerobic medium, prepared tryptic soy broth (TSB) and tryptic soy agar (TSA) media were gassed with N_2_ under the atmospheric concentration. The media were dispensed into 18 mm × 150 mm anaerobic tubes (4 mL/tube) under N_2_ stream and sealed with butyl rubber stoppers. These tubes were sterilized in an autoclave for 20 min (Kwon and Ricke, 1998). Pure anaerobic isolates were maintained on TSA plates kept in an anaerobic chamber (ELECTROTEK anaerobic chambers, AW300SG, Britain) with filtered anaerobic gas mixture (80% N_2_, 10% CO_2_, and 10% H_2_).

### Chemicals

In the species specific verbenone conversion experiments, TSA and TSB medium were purchased from Sigma-Aldrich (Shanghai, China). (S)-*cis*-verbenol (95% purity), (1S)-(-)-verbenone (94% purity) and heptyl acetate (≥98% purity) were purchased from Sigma-Aldrich (Shanghai, China) for use in all experiments.

### Isolation and Identification of Bacteria

The guts from selected beetles were dissected under anaerobic conditions. Individual guts from adult beetles (*n* = 120) were ground for 10 s in 200 μl of 10% PBS (phosphate buffer saline solution) and the suspension spread evenly on TSA medium. The dilution factors varied from 10^2^ to 10^6^. After incubation in anaerobic chamber at 28°C for 30 days, colonies of pure bacterial cultures from each sample were selected and streaked. The combination of thickness size, color and size etc. were morphologically categorized and counted for pure cultures.

Samples of isolates were selected for 16S rDNA sequencing using DNeasy Blood Kit for extracted DNA (Qiagen, Germany). The 16S ribosomal RNA genes were amplified with primers 8F (5′-GCGGATCCGCGGCCGCTGCAGAGTTTGATCCTGGCTCAG-3′) and 1492R (5′-GGCTCGAGCGGCCGCCCGGGTTACCTTGTTACGACTT-3′) (Weisburg et al., 1991). PCR reactions were performed on an Eppendorf Mastercycler Gradient (Eppendorf, Germany). The reaction mixture contained 1.2 μl of dNTPs (10 mM each), 5 μl of 10× PCR buffer (with MgCl_2_), 2 μl of primers (10 μM each), 0.8 μl Taq polymerase (5 U/μl) (TaKaRa, China) and 10–100 ng of DNA adjusted to 50 μl with sterilized deionized water. The reaction conditions were 94°C for 5 min; 35 cycles of 30 s at 94°C, 30 s at 51°C, and 1 min and 30 s at 72°C and a final extension at 72°C for 10 min. PCR products with expected size of 1500 bp were visualized on 1% agarose gels and purified by Axygen DNA Gel Extraction Kit (Axygen, United States). The samples were sequenced in two directions on an ABI 3730XL DNA Analyzer (Applied Biosystems, United States) using the same primers. Consensus sequences were manually assembled and edited according to chromatograms in MEGA5 (Tamura et al., 2011). Alignments were done online using the EzTaxon-e server^1^ (Kim et al., 2012) and BLAST search^2^. Sequences in this study were deposited in the GenBank database (**Table 1**).

**Table 1:** Frequently identified isolates of *Dendroctonus valens* gut associated facultative bacteria.

Isolate numbers	Accession numbers	Closest type strains and ecologically related strains	Species affiliation	Similarity (%)
			**Proteobacteria**	
			**Enterobacteriaceae**	
B3	MF083086	*Klebsiella michiganensis* W14(T)	*Klebsiella michiganensis*	99
B1	MF083087	*Enterobacter xiangfangensis* 10-17(T)	*Enterobacter xiangfangensis*	100
			**Erviniaceae**	
B6	MF083088	*Pantoea conspicua* LMG 24534(*T*)	*Pantoea conspicua*	99
B2	MF083081	***Erwinia* sp. FJ811869 (*Dendroctonus valens*)**	*Erwinia sp.*	99
			**Yersiniaceae**	
B11	MF083085	*Serratia liquefaciens* ATCC 27592(T)	*Serratia liquefaciens*	99
B7	MF083084	*Rahnella variigena* CIP 105588(T)	*Rahnella variigena*	99
B8	MF083083	***Rahnella aquatilis* KJ781940 (*Dendroctonus valens*)**	*Rahnella aquatilis*	99
			**Firmicutes**	
			**Lactobacillaceae**	
B4	MF083089	*Lactobacillus acidophilus* CIP 76.13(T)	*Lactobacillus acidophilus*	100
			**Staphylococcaceae**	
B13	MF083080	*Staphylococcus epidermidis* ATCC 14990(T)	*Staphylococcus epidermidis*	99
B12	MF083082	*Staphylococcus warneri* ATCC 27836(T)	*Staphylococcus warneri*	99

*GenBank accession numbers of isolates from adult Dendroctonus valens guts, and similarity scores to closest type strains and ecologically related strains in NCBI according to the 16S rDNA. “T” indicates type strain and strain in bold indicate ecologically related strain.*

### Phylogenetic Analyses

The identical sequences of the 10 bacterial isolates were subjected to phylogenetic analysis. An additional 35 sequences from the two databases mentioned above, most of which were of type strains and ecologically related strains, were added using Clustal X (Thompson et al., 1997) followed by manual refinement in BIOEDIT (Hall, 1999). jModelTest 2.1 (Darriba et al., 2012), which showed that the GTR+I+G model was the most appropriate model according to the Akaike information criterion (Posada and Buckley, 2004). The phylogeny was constructed by the maximum likelihood approach using RAxML version 7.4.2 (Stamatakis et al., 2004). Confidence at each node was assessed by 1000 bootstrap replicates (Hillis and Bull, 1993). The resulting tree was visualized and edited with Tree Graph 2 (Stöver and Müller, 2010) and refined with Adobe Illustrator CS3. *Anabaena affinis* (AF247591) was considered as an out group.

### Conversion Experiments

To calculate the *cis*-verbenol concentration of beetle’s hindgut, we followed the methodology described by Vasanthakumar et al. (2006) to determine the ratio of the amount of *cis*-verbenol in a beetle’s hindgut (10^0^ to 10^3^ ng) to the estimated hindgut volume (1.21 ± 0.48 μl). The *cis*-verbenol concentration in a beetle’s hindgut was estimated in a range from 10^0^ to 10^2^ ng/μl, hence the concentrations of 4, 40, and 200 ng/μl selected for these experiments. All of the 10 bacterial isolates were grown in TSB medium and incubated for 24 h. A dilution of 1:100 of each isolate was made when cultures were adjusted to an optical density (OD_600_) of 0.5. Each concentration of *cis*-verbenol was then added into 4 ml bacterial suspension and shaken for a further 36 h. A suspension containing an equivalent amount of *cis*-verbenol without bacteria was run as a control in the same manner for each bacterial species tested. All solutions were extracted with hexane and then stored for later chemical analysis to examine verbenone concentration. The conversion experiments followed previosly described methods (Xu et al., 2015), except for incubation and shaking times.

### Measurement of O_2_ Concentrations in Beetle Hindgut and Frass

Dispersing female and male adult beetles (sexes were distinguished by listening to stridulations produced by males) were captured with the standard *D. valens* lure [(+)-α-pinene: (+)-β-pinene: (+)-3-carene = 1: 1 : 1] in Beishe and Laoyagou mountains of Shanxi province during June 2017. To obtain colonizing beetles, logs (diameter ≥ 30 cm, lengths° = 50 cm) were cut from healthy trees and three pairs of adult beetles were introduced to each bolt (four bolts). The ends of the bolts were sealed with wax to keep in moisture, then immediately transported to the laboratory and the beetles allowed to colonize the bolt for a period of 1 month. Of the trapped dispersing beetles, 10 of the healthiest male and female adults were selected for O_2_ concentration measurement in their hindgut. While for colonizing beetles, 10 males and females were extracted from the bolts. The hindguts of both the female and male adults were extracted and placed on a 2 mm thick layer of 1.5% agarose and then covered with a 2 mm layer of 0.5% agarose. Clark-type oxygen microelectrodes (tip diameters of 10 mm, 90% response times of <3 s, and stirring sensitivities of <2%) containing guard cathodes were constructed in our laboratory were used for the measurement of O_2_ concentrations. Prior to use, the electrodes were polarized for 12 h in deionized water that was continuously bubbled with air. They were calibrated by measuring the current when the microelectrode was placed in water saturated with air (21% O_2_), as well as the background current in water sparged with 100% N_2_ (0% O_2_). Calibration was carried out before and after each experiment. The current was measured with a picoammeter (model 1201; Diamond General, Ann Arbor, MI, United States) connected to a strip chart recorder. For O_2_ concentration measurements in frass, microelectrodes used had a tip diameter of 20 mm. Ten pairs of adults were introduced to fresh pine logs for a period of 1 month and the guts of the colonizing beetles (from beetles extracted from the pine logs) were analyzed for O_2_ concentration. The microelectrode measurements methods described in Brune et al. (1995) were followed, except for O_2_ concentrations measurements of frass, where microelectrodes were directly probed into the frass *in situ*.

### Comparative Conversion Experiments in Different Oxygen Environments

Three of the most active bacterial isolates of *D. valens* gut that converted significantly higher amounts of *cis*-verbenol to verbenone in the previous experiments were selected to test the conversion efficiency under anaerobic conditions, 10% (80% N_2_, 10% CO_2_, and 10% O_2_) and 20% (80% N_2_ and 20% O_2_) oxygen concentrations, respectively. A suspension containing an equivalent amount of *cis*-verbenol without bacteria was run as a control in the same manner for each bacterial species tested. The conversion experiments followed previosly described methods.

### Chemical Analysis

The concentrations of verbenone produced during each bio assay were determined using gas chromatography-mass spectrometry. Two microliter extracts were injected, splitless, into GC-MS (Agilent 6980N GC coupled 5973 mass selective detector) equipped with an HP5-MS capillary column (0.25 mm i.d. × 60 m; Agilent Technologies, Inc., Palo Alto, CA, United States) and the column temperature was programmed from an initial temperature of 50°C for 1 min, then ramped 5°C/min to 100°C, by 3°C/min to 130°C and by 20°C to 320°C (2 min hold time). Components of the extracts were identified by comparing retention times and mass spectra with authentic standards from NIST 02 library (Scientific Instrument Services, Inc., Ringoes, NJ, United States). Quantification was performed using an internal standard (heptyl acetate) that was added to each sample.

### Statistical Analysis

This study used the Scheirer–Ray–Hare test as variances were unequal even after initial data transformation (Dytham, 2011), and Dunnett’s T^3^ test was used for *post hoc* comparisons. All data were analyzed using SPSS 12.0 (SPSS Inc., Chicago, IL, United States).

## Results

### Isolation and Identification of Bacterial Species

**FIGURE 1:**
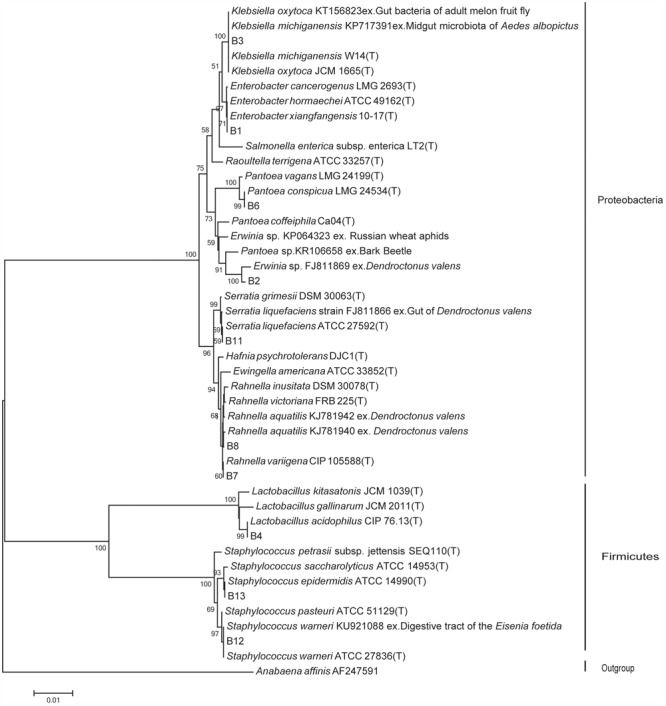
Maximum likelihood tree for the 16S rRNA of *Dendroctonus valens* gut associated facultative bacteria. The ecologically related (indicated after the accession number with “ex.” as the beginning) sequences from GenBank and the types (indicated with “T”) from EzTaxon-e database. The 16S rRNA sequence of *Anabaena affinis* was used as outgroup. Numbers on the nodes represent bootstrap support from 1,000 replicates. Nodes with bootstrap values of 50% or more are indicated.

A total of 652 gut facultative bacterial species were isolated from gut samples purified from 120 adult *D. valens* beetles (dispersing male, dispersing female, colonizing female, colonizing male, and their phylogeny). BLAST results and phylogenetic analyses were used to identify the isolated species. The results confirmed 10 species of gut facultative bacterial belonging to a total of five families [(viz. Enterobacteriaceae, Erviniaceae, Yersiniaceae, Lactobacillaceae, Staphylococcaceae) and two phyla (viz Proteobacteria (7 species) and Firmicutes (3 species))] (**Table 1** and **Figure 1**).

**FIGURE 2:**
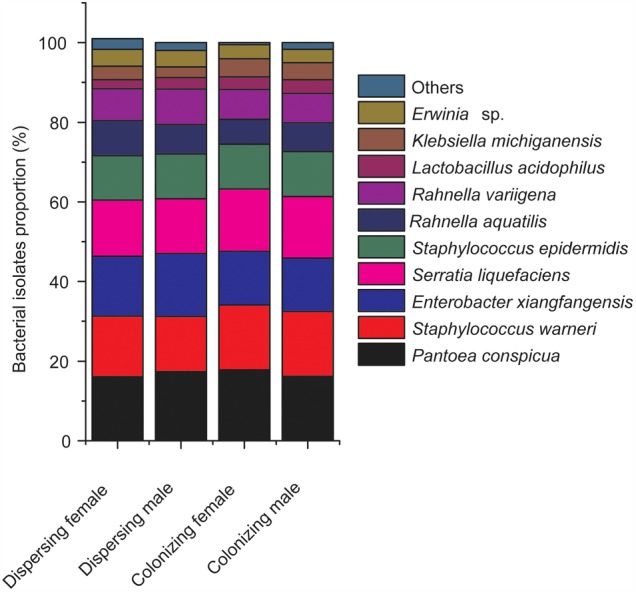
Composition and relative proportion of *Dendroctonus valens* gut associated facultative bacteria. Species with fewer than 2% were pooled as others.

Among the identified species, the most frequently isolated species were *Pantoea conspicua* (dispersing female, 16.1%; dispersing male, 17.4%; colonizing female, 17.8%; and colonizing male, 16.2%), *Staphylococcus warneri* (dispersing female, 15.2%; dispersing male, 13.8%; colonizing female, 16.3%; and colonizing male, 16.3%), *Enterobacter xiangfangensis* (dispersing female, 15%; dispersing male, 15.8%; colonizing female, 13.5%; and colonizing male, 13.4%) (**Figure 2**). The remaining species accounted for less than 52% in both sexes of *D. valens* adults in the two life history stages. *Lactobacillus acidophilus* was the least frequently isolated bacterial species.

### *cis*-Verbenol – Verbenone Conversion

**FIGURE 3:**
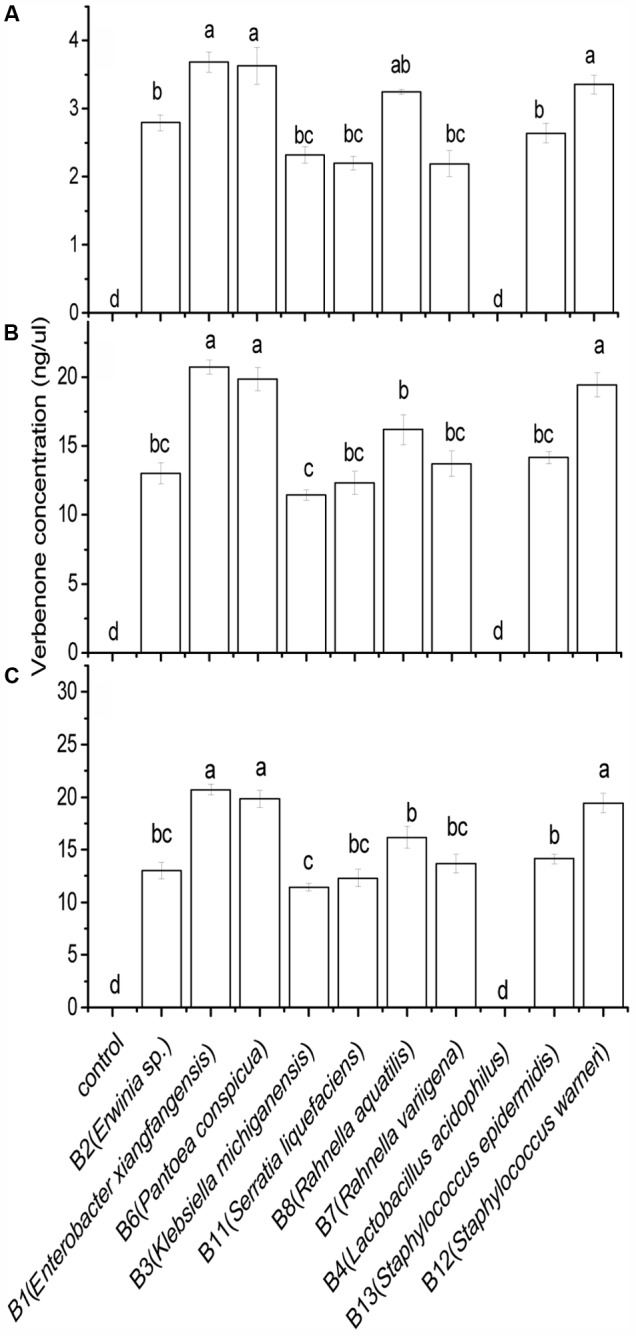
Verbenone production by *Dendroctonus valens* gut associated facultative bacteria under anaerobic environment. The amounts of verbenone produced by facultative bacteria at **(A)** 4 ng/μl, **(B)** 40 ng/μl, and **(C)** 200 ng/μl *cis*-verbenol concentrations. Statistical analysis was performed using Dunnett’s T^3^ test. The bars with different letters are significantly different at *P* = 0.05.

The results of the Scheirer–Ray–Hare test revealed a significant association between bacterial isolates and *cis*-verbenol concentrations in terms of pheromone production in *D. valens* (**Table 2**). The amount of verbenone produced by *D. valens* in association with its gut-associated facultative bacterial isolates at three concentrations (4, 40, and 200 ng/μl) of *cis*-verbenol is shown in **Figure 3**. Nine out of the 10 isolates had resulted into significantly higher concentrations of verbenone compared to control, while no verbenone was detected in facultative bacterial species B4 (*L. acidophilus*). The highest level of *cis*-verbenol conversion ability was observed in B1 (*E. xiangfangensis*), B6 (*P. conspicua*), and B12 (*S. warneri*), representing the three most frequently isolated species in *D. valens* guts. Incubation of B1 (*E. xiangfangensis*) with three concentrations of *cis*-verbenol yielded 3.68 ± 0.14, 20.18 ± 0.75, and 20.71 ± 0.51 ng/μl of verbenone; B6 (*P. conspicua*) with three concentrations of *cis*-verbenol yielded 3.62 ± 0.27, 19.54 ± 9.07, and 19.83 ± 9.20 ng/μl of verbenone; B12 (*S. warneri*) with three concentrations of *cis*-verbenol yielded 3.35 ± 0.13, 19.33 ± 0.79, and 19.43 ± 0.89 ng/μl of verbenone, respectively.

**Table 2:** Statistics for association between facultative bacteria and pheromone *cis-*verbenol concentration using Scheirer–Ray–Hare test.

Source	*df*	SS	*H*	*P*-value
*cis*-Verbenol concentrations	2	60720	8.23	<0.001
Bacterial isolates	9	14093	1.91	<0.001
Bacterial isolates × *cis*-verbenol	18	1108	0.15	<0.001
concentrations				

*cis-Verbenol concentrations as independent variables and verbenone production as dependent variable. df, degrees of freedom; SS, sum of squares; H, Scheirer–Ray–Hare non-parametric two-way analysis of variance statistic.*

### Measurement of O_2_ Concentrations in Beetle Hindgut and Frass

**FIGURE 4:**
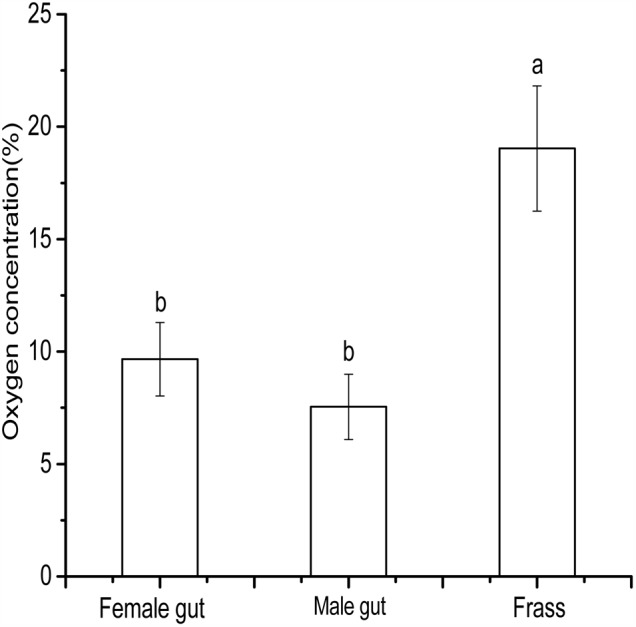
Oxygen concentrations of *Dendroctonus valens* gut and frass. The bars with different letters are significantly different at *P* = 0.05.

The highest (19.03%) O_2_ concentration occurred in frass of *D. valens*, while the hindgut of male and female beetles had lower O_2_ concentrations (7.54 and 9.65%, respectively) that were statistically similar but significantly lower than that in the frass (**Figure 4**). The oxygen concentration in frass was closer to the atmospheric O_2_ concentration, whereas the average O_2_ concentration of gut female and gut male oxygen concentration 8.59%, which means the gut is an oxygen-limited micro-environment. These results are very important to evaluate the relevance of the oxygen concentrations used in the conversion experiments.

### Comparative Conversion Experiments in Different Oxygen Environments

**FIGURE 5:**
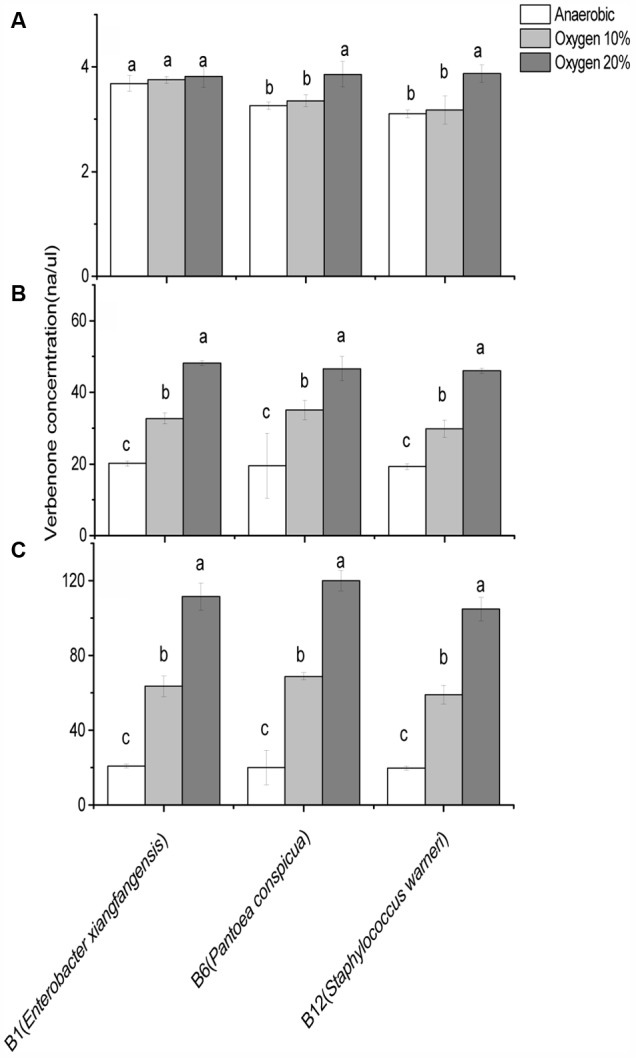
Comparison of *cis*-verbenol to verbenone conversion ability of *Dendroctonus valens* gut associated facultative bacteria under different oxygen concentrations. Three bacteria from *Dendroctonus valens* gut are compared in terms of their *cis*-verbenol to verbenone conversion ability at **(A)** 4 ng/μl, **(B)** 40 ng/μl, and **(C)** 200 ng/μl *cis*-verbenol concentrations. Statistical analysis was performed using Dunnett’s T^3^ test. The bars with different letters are significantly different at *P* = 0.05.

In the previous experiments, B1 (*E. xiangfangensis*), B6 (*P. conspicua*), and B12 (*S. warneri*) isolates of gut bacteria produced the highest concentrations of verbenone, hence these three species were selected to test the oxygen dependent verbenone production. The experiments were conducted under three oxygen concentrations (anaerobic, 10% and 20%) and with three concentrations of *cis-*verbenol (4, 40, and 200 ng/μl). Anaerobic and 10% oxygen concentration was selected as gut-simulated oxygen concentration while the oxygen concentration of 20% was selected as frass-simulated concentration. The results are presented in **Figure 5**.

In general, significantly higher concentrations of verbenone were recorded under 20% oxygen concentration compared to the 10% oxygen and anaerobic conditions. Whereas, when 40 and 200 ng/μl concentrations of *cis*-verbenol were offered for conversion, significantly more conversion was observed under 10% oxygen than the anaerobic conditions. In terms of bacterial efficiencies regarding verbenone production, no significant difference was observed at different oxygen concentrations when B1 (*E. xiangfangensis*) bacterial species was incubated with 4 ng/μl concentrations. Whereas, B6 (*P. conspicua*) and B12 (*S. warneri*) converted significantly higher amounts of *cis-*verbenol to verbenol at 20% oxygen concentrations when incubated at 4 ng/μl concentrations of *cis*-verbenol.

## Discussion

Oxygen is an important physicochemical factor that not only is required for survival of insect gut-associated aerobic bacteria but also influences their function by altering the chemical milieu of gut lumen. Hence the composition of gut-associated bacterial community and their functions vary with different levels of oxygen concentration (Brune et al., 1995). In general, the oxygen levels in the gut of herbivorous insects is considered a low oxygen environment (Johnson and Barbehenn, 2000), and little is known about oxygen levels in the gut lumen and frass of bark beetles. Thus, we initially used clark-type oxygen microelectrodes to obtain high-resolution profiles of oxygen concentrations in isolated guts and frass of *D. valens* and confirmed that the gut (8.59%) of *D. valens* is not a strict anoxic environment and frass (19.03%) contains an oxygen-rich environment, hence supporting our finding that no obligate anaerobic bacteria existed there. Thus, we designed experiments to evaluate the effect of gut-simulated and frass-simulated oxygen concentration environment on *cis*-verbenol to verbenone conversion efficiency of gut associated bacteria by comparing their activities under different oxygen concentrations.

This is the first report of gut bacterial species isolated and identified from *D. valens* from an anaerobic environment. One of the frequent bacterial species isolated was *P. conspicua* which is an enteric bacterium ubiquitous in bark beetle’ s gut and other general environment (Vasanthakumar et al., 2006; Delalibera et al., 2007; Morales-Jimenez et al., 2009; Nadarasah and Stavrinides, 2014), followed by *E. xiangfangensis* and *S. warneri*, all of which are common insect gut associates (Harada et al., 1996; Podgwaite et al., 2013; Manfredi et al., 2015) including bark beetles (Morales-Jimenez et al., 2009), e.g., *P. conspicua* has been found in Mexican *Dendroctonus valens* (19.23% abundance) using culture-independent analysis (Morales-Jimenez et al., 2009). In general, facultative anaerobic or microaerophilic cellulolytic bacteria were often isolated in aerobic cultivation conditions from the gut of insects (Wenzel et al., 2002; Hernández et al., 2015). However, the genera *Pantoea, Enterobacter*, and *Staphylococcus* have not been reported in *D. valens* gut in aerobic environment (Xu et al., 2015). This discrepancy might be due to aerobic bacteria growing better than facultative anaerobic bacteria, which explains the lack of isolates of facultative anaerobic or anaerobic bacteria in aerobic environments. Our results showed that no obligate anaerobic bacteria was found in the gut of *D. valens*, which agrees with reports in *Dendroctonus frontalis* and *Ips pini* (Vasanthakumar et al., 2006; Delalibera et al., 2007).

A previous study used 16S rRNA gene sequencing to survey gut microbial community of *Dendroctonus valens* and structure and abundance of different microbiota (Xu et al., 2016b), while the focus of the current study is a continuation to further compare their ability to convert *cis*-verbenol into verbenone (a multi-functional pheromone of *D. valens*) under different O_2_ concentrations. Hence, a culture-dependant method is necessitated to create experimental conditions of a variable oxygen environment. We have attempted to bridge the knowledge gap (lack of species ID, and inability to vary oxygen concentration) that exists between using culture-independent (16S rRNA) and culture-dependent methods in order to make up the shortfall and solve the research problem. To better illustrate the methodological differences, we created a table (Supplementary Table S1) to compare the gut bacteria and their abundance within each group between the two methods. The composition and abundance of microbiota in insect guts change dynamically in relation to insect feeding (Mason and Raffa, 2014) and physicochemical factors (Engel and Moran, 2013). In addition, there was a large difference in the abundance between female gut and male gut bacteria in 16S rRNA sequencing (Xu et al., 2016b), and differences are apparent when comparing the different identification methods.

The more abundant gut bacteria might not necessarily be involved in the synthesis of pheromones in the gut environment; rather it could be the nitrogen-fixation bacteria (Morales-Jimenez et al., 2009) or biodegradable defense bacteria (Dillon and Dillon, 2004; Engel and Moran, 2013; Manfredi et al., 2015). On the other hand, the gut bacteria of low abundance species might be the key species to convert *cis*-verbenol to verbonene. Although culture-independent methods (16S rRNA sequencing) provide an unbiased approach, it could only identify the bacterial genus but couldn’t positively identify the particular bacterial species or strain. Furthermore, the method doesn’t allow manipulation of experimental conditions with individual strains. Other factors such as human errors, sampling or culturing methods can also resulted in the difference in abundance or absence of some strains. The gut is a low oxygen environment under normal circumstances (mean is 8.49% oxygen), and an anaerobic gut environment or oxygen rich environment cannot be controlled for *in situ*. Even if we could artificially vary the gut’s oxygen environment, it may have limited biological significance since these conditions are far outside the observed normal range.

Furthermore, we have investigated the role of oxygen on facultative anaerobes in the chemical ecology of *D. valens.* The beetles communicate using bioactive gut volatile and verbenone (Zhang et al., 2006; Sun et al., 2013). Verbenone production in *D. valens* mainly depends upon the rate of conversion of *cis*-verbenol and *trans*-verbenol to verbenone and this process is proven to be facilitated by beetles’ gut-associated bacteria (Xu et al., 2015). Earlier studies on pheromone conversion had not taken into account the actual oxygen concentrations in the gut, and instead were performed at atmospheric O_2_ concentrations. Hence, we have demonstrated the ability of isolated species to convert *cis-*verbenol into verbenone under anaerobic–aerobic conditions. Thus, until it’s possible to vary the O_2_ concentrations in the guts *in situ*, the current assessment using a culture dependent is a more reasonable representation of the lower oxygen level in *D. valens* gut.

It has been clearly observed that the majority (ca. 90%) of the isolates were able to convert *cis-*verbenol to verbenone in a gut-simulated oxygen environment and their conversion efficiencies increased with increasing oxygen concentration. This may be partly explained by physicochemical influence of O_2_ on the production of reactive oxygen species, where increased concentration of oxygen results in an increase in the amount of reactive oxygen species (Badr et al., 1989). Another possibility is that facultative bacteria have different growth rates in the environments with two distinct oxygen concentrations since oxygen tensions has already been known to inhibit or delay the growth of many aerobic or facultative anaerobic bacteria (Moore and Williams, 1911). Whether the variation of pheromone production between the three oxygen concentration environments are caused by physicochemical influence of oxygen, different growth rates or a combination of both, requires further exploration.

Previous studies of the function of gut bacteria are mostly conducted under atmospheric conditions (Xu et al., 2015; Yuki et al., 2015). In this study, the pheromone conversion efficiency by the bacteria under the gut-simulated and frass-simulated oxygen concentration environment more accurately reflects actual pheromone conversion efficiency in *D. valens* gut and frass, and our findings strongly suggest that oxygen concentration is a significant factor influencing pheromone production facilitated by facultative anaerobic bacteria. Since the frass of *D. valens* has been found to be a rich O_2_ environment, this suggests that the facultative anaerobic bacterial species capable of verbenone production can exist there. Frass appears to play a more important role in the process of attacking and the behavior regulation in the wild than previously thought, implying its important ecological significance. Future work should consider factors affecting function of gut bacteria, such as hydrogen and pH, and studies should also involve the functions of these facultative anaerobes in *D. valens* development, detoxification, and chemical communication, which will lead to better understanding of the complex symbiotic relationship of bark beetles with microorganisms.

## Author Contributions

JS and ML conceived and designed the experiments. QC and LC performed the experiments and analyzed the data. QC, JW, and FA wrote the paper.

## Conflict of Interest Statement

The authors declare that the research was conducted in the absence of any commercial or financial relationships that could be construed as a potential conflict of interest.

## Footnotes

1 http://www.eztaxon.org/

2 http://blast.ncbi.nlm.nih.gov/Blast.cgi
