# Supplementary material for: Effect of Oxygen on Verbenone Conversion From cis-Verbenol by Gut Facultative Anaerobes of Dendroctonus valens
Source: Front Microbiol. 2018 Mar 16;9:464. doi: 10.3389/fmicb.2018.00464 (PMC5864928; doi:10.3389/fmicb.2018.00464)
Supplement: Supplementary file 1 [file Table_1.doc]

**Effect of oxygen on verbenone conversion from *cis*-verbenol by gut facultative anaerobes of *Dendroctonus valens***

**Qingjie Cao^1 #^ , Jacob Wickham ^1 #^, Li Chen^1,2^ , Faheem Ahmad^3^ , Min Lu^1^* , Jianghua Sun^1,2^***

^1^State Key Laboratory of Integrated Management of Pest Insects and Rodents, Institute of Zoology, Chinese Academy of Sciences, Beichen West Road, Chaoyang District, Beijing, China.

^2^University of Chinese Academy of Sciences, Beijing, China.

^3^Department of Biosciences, COMSATS Institute of Information Technology, Park Road, Chak Shehzad, Islamabad, Pakistan.

**#** [Equal](../../../../C:%5CUsers%5CAdministrator%5CAppData%5CLocal%5Cyoudao%5Cdict%5CApplication%5C7.5.0.0%5Cresultui%5Cdict%5C%3Fkeyword=equal&lang=en) Contribution

*** Correspondence:** [lumin@ioz.ac.cn](mailto:lumin@ioz.ac.cn)**,** sunjh@ioz.ac.cn

**Number of supplementary table:** 1

**Supplementary Table S1: Comparison of identification of *Dendroctonus valens* gut associated bacteria and their average abundance (mean ± SEM) within each group under different methods.**

| Phylum | Culture-independent (16S rRNA) | | | Culture-dependent | |
| --- | --- | --- | --- | --- | --- |
|  | Phylogenetic group (Genus) | Female gut% | Male gut% | Phylogenetic group (Species) | Gut % |
| Firmicutes | *Lactobacillus*** | 0.11 ± 0.03 | 0.07 ± 0.02 | *Lactobacillus acidophilus* | 2.97 ± 0.47 |
|  | *Staphylococcus** | 0.008 ± 0.0029 | | *Staphylococcus epidermidis* | 11.25 ± 0.08 |
|  |  |  |  | *Staphylococcus warneri* | 15.40 ± 1.09 |
| Proteobacteria | *Rahnella*** | 0.07 ± 0.01 | 1.34 ± 1.08 | *Rahnella variigena* | 7.90 ± 0.68 |
|  |  |  |  | *Rahnella aquatilis* | 7.42 ± 0.95 |
|  | *Klebsiella** | 0.001 ± 0.0004 | | *Klebsiella michiganensis* | 3.75 ± 0.80 |
|  | *Enterobacter* |  |  | *Enterobacter xiangfangensis* | 14.42 ± 0.83 |
|  | *Pantoea*** | 0.02 ± 0.01 | 0.31 ± 0.15 | *Pantoea conspicua* | 16.87 ± 0.79 |
|  | *Erwinia*** | 52.02 ± 11.00 | 56.77 ± 6.06 | *Erwinia sp.* | 3.77 ± 0.40 |
|  | *Serratia** | 0.002 ± 0.0008 | | *Serratia liquefaciens* | 14.72 ± 0.83 |

* indicates identity of the genera shared and their average abundance (mean ± SEM) observed from Xu et al. (2016c, see supplementary materials); ** indicates identity of the genera shared and their average abundance observed (mean ± SEM) from Xu et al. (2016b).

**References**

Xu, L., Lu, M., Xu, D., Cheng, L.,and Sun, J. (2016b). Sexual variation of bacterial microbiota of *Dendroctonus valens* guts and frass in relation to verbenone production. *J Insect Physiol* 95,110-117.doi.org/10.1016/j.jinsphys.2016.09.014.

Xu, L., Shi Z., Wang, B., L., Lu, M., and Sun, J. (2016c). Pine Defensive Monoterpene a-Pinene Influences the Feeding Behavior of *Dendroctonus valens* and Its Gut Bacterial Community Structure. *Int J Mol Sci* 17(11),1711-1734.doi:10.3390/ijms17111734.
